# Improvement of Bone Physiology and Life Quality Due to Association of Risedronate and Anastrozole

**DOI:** 10.3389/fphar.2017.00632

**Published:** 2017-09-12

**Authors:** Vincenzo Monda, Gelsy A. Lupoli, Giovanni Messina, Rosario Peluso, Annalisa Panico, Ines Villano, Monica Salerno, Francesco Sessa, Francesca Marciello, Fiorenzo Moscatelli, Anna Valenzano, Leonardo Molino, Roberta Lupoli, Francesco Fonderico, Anna Tortora, Agata Pisano, Maria Ruberto, Marsala Gabriella, Gina Cavaliere, Giovanna Trinchese, Maria P. Mollica, Luigi Cipolloni, Giuseppe Cibelli, Marcellino Monda, Giovanni Lupoli, Antonietta Messina

**Affiliations:** ^1^Section of Human Physiology and Unit of Dietetic and Sport Medicine, Department of Experimental Medicine, University of Campania “L. Vanvitelli” Naples, Italy; ^2^Department of Clinical Medicine and Surgery, University of Naples Federico II Naples, Italy; ^3^Department of Clinical and Experimental Medicine, University of Foggia Foggia, Italy; ^4^Rheumatology Research Unit, University of Naples Federico II Naples, Italy; ^5^Department of Oncohematology, Santa Maria delle Grazie Hospital Pozzuoli, Italy; ^6^Department of Medical-Surgical and Dental Specialties, University of Campania “L.Vanvitelli” Naples, Italy; ^7^Struttura Complessa di Farmacia, Azienda Ospedaliero-Universitaria, Ospedali Riuniti di Foggia Foggia, Italy; ^8^Department of Biology, University of Naples Federico II Naples, Italy; ^9^Università degli Studi di Roma La Sapienza Rome, Italy

**Keywords:** health-related quality of life, bone density, anastrozole, risedronate, breast cancer

## Abstract

The endocrine therapy is the new frontiers of many breast cancers hormone sensitive. Hormone therapy for treating women with hormone receptor-positive cancer suppresses breast cancer growth either by reducing estrogen synthesis or by interfering with the action of estrogen within tumor cells. In this prospective randomized observational study we investigate the effect of adjuvant anastrozole in monotherapy or associated with risedronate on bone physiology and quality of life in postmenopausal, hormone-sensitive early breast cancer women at mild to moderate risk of fragility fractures.

**Methods :** 84 women were randomly assigned to receive anastrozole alone (group A) or anastrozole plus oral risedronate (group A+R). At baseline and after 24 months lumbar spine (LS) and femoral neck (FN) BMD were evaluated with dual-energy x-ray absorptiometry and health-related quality of life (HRQoL) was examined using the short-form healthy survey.

**Results :** After 24 months, the group A+R has showed a significant increase in T-score for LS (*p* < 0.05) and for FN (*p* < 0.05) whereas women of group A had a statistically significant rate of bone loss both in LS T-score (*p* < 0.05) and in FN (*p* < 0.05). A significant change in T-score BMD was seen for group A+R compared with group A at the LS (*p* = 0.04) and at FN (*p* = 0.04). Finally, group A+R showed an overall significant improvement of health profile (SF-36) in group A (*p* = 0.03).

**Conclusion :** Postmenopausal breast cancer women with osteopenia during treatment with anastrozole have considerable risk of developing osteoporosis during the first 2 years; preventive measures such as healthy lifestyle and daily supplements of calcium and vitamin D alone seem to be insufficient in holding their bones healthy. Our findings suggest the usefulness of addition of risedronate in order to prevent aromatase inhibitors-related bone loss, not only in case of high-risk of fractures, but also for women at mild-moderate risk. This determines a significant improvement in bone health and a positive impact on HRQoL.

## Introduction

The endocrine therapy is the new frontiers of many breast cancers hormone sensitive, linked to specific genetic alteration such as copy number aberrations (i.e., *ERBB2, CCND1* amplification loci), homozygous deletions of *CDKN2A/B* and *PTEN*, and high-frequency substitution and insertion/deletion (indel) driver mutations in cancer genes like *TP53* (~frequency 53%), *PIK3CA* (8–26%), *CDH1* (21%), *AKT1* (8%) and *GATA3* (4%). Moreover, germline exploration has led to documentation of rare, high-penetrance (*BRCA1, BRCA2, TP53*), moderate penetrance (*PTEN, STK11, CDH1, ATM, CHEK2, BRIP1, PALB2*) and common, low penetrance risk alleles for developing breast cancer (Li Volti et al., 2011; Salomone et al., 2013; Tibullo et al., 2013; Pomara et al., 2016; Barone et al., 2017; Hahnen et al., 2017; Nik-Zainal and Morganella, 2017; Rizvi et al., 2017). Hormone therapy for treating women with hormone receptor-positive cancer suppresses breast cancer growth either by reducing estrogen synthesis or by interfering with the action of estrogen within tumor cells (Gradishar and Jordan, 1999). In postmenopausal women, the treatment of breast and ovarian cancer are carried out with aromatase inhibitors (AIs); they effect on the peripheral conversion of androgens to estrogens, acting on cytochrome P450 CYP-19 enzyme (Smith and Dowsett, 2003). The adjuvant treatment of postmenopausal women with hormone receptor-positive early breast cancer was carried out with third generation of AIs, such as anastrozole, exemestane and letrozole that have shown superior efficacy and generally better safety and tolerability profile compared with tamoxifen (Arimidex, 2008). However, the bone metabolism is influenced indirectly and positively by estrogens. Stimulating the production of several cytokines, they act either as inhibitors of osteoclastogenesis or as antireceptive agents leading active osteoclasts to apoptosis: several clinical studies have demonstrated the effects of AIs on bone physiology showing an increase in biochemical markers of bone turnover, a decrease in bone mineral density (BMD) and a consequent increase of risk of fractures (Eastell and Hannon, 2005; Santen, 2011; Di Bernardo et al., 2014; Triggiani et al., 2015). Moreover, skeletal fragility and fracture risk are increased by breast cancer, even in the absence of bone metastases. The secretion of the parathyroid hormone-related protein, the chemotherapy, or premature ovarian failures can mediate these effects (Perez and Weilbaecher, 2006; Marra et al., 2013; Monda et al., 2014; De Fusco et al., 2017; Panico et al., 2017); estrogen deprivation caused by AIs administration can exacerbate this risk. To avoid this adverse event, antiresorptive agents such as bisphosphonates (BPs) can be used in breast cancer and in combination with AIs (Greenspan et al., 2007; Hines et al., 2009; Van Poznak et al., 2010; Messina et al., 2017). Bisphosphonates are pyrophosphate analogs which contain a phosphate-carbon-phosphate (P-C-P) core structure. They have an effect on bone escaping from enzymatic degradation. Risedronate is a pyridinyl bisphosphonate validated for the prevention and treatment of postmenopausal osteoporosis (Valenzano et al., 2016). His efficacy was described in decreasing the rate of bone turnover and reversing the loss of BMD, with an adverse effect profile that is similar to placebo.

In this study we investigate the effect of adjuvant anastrozole in monotherapy or associated with risedronate on bone physiology and quality of life in postmenopausal, hormone-sensitive early breast cancer women having pre-existing mild to moderate risk of fragility fractures.

## Materials and methods

**Figure 1:**
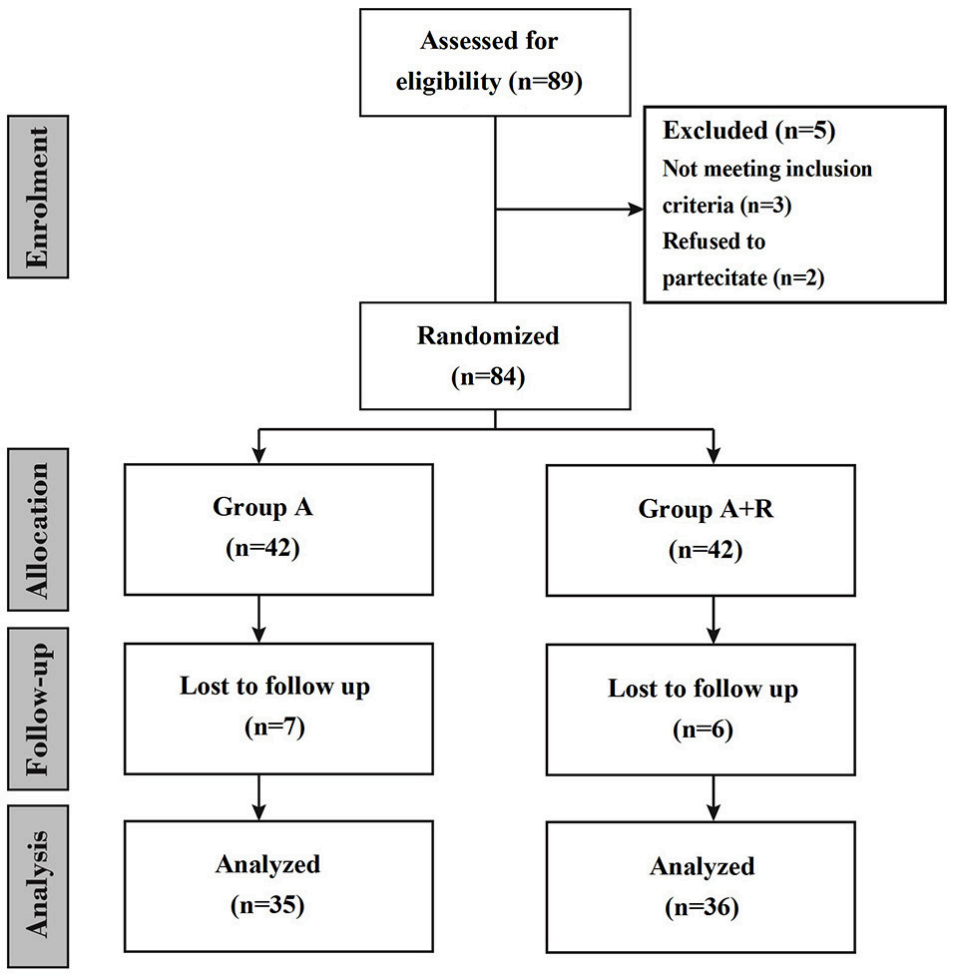
Flow chart of the study.

This is a prospective randomized observational study in which women were treated for 24 months (Figure 1). Participants were provided with both written and oral information regarding the study protocol and were ensured that they were free to withdraw from the study at any time. Before participation, informed consent were subscribed. All procedures conformed to the directives of the Declaration of Helsinki. This study has been approved by the Azienda Universitaria Policlinico (AUP) of the University of Naples. We enrolled a total of 84 eligible postmenopausal women with hormone receptor-positive breast cancer; they had completed primary surgery and chemotherapy (if indicated) and were categorized according to their baseline BMD T-score as affected by osteopenia (T-score less than–1.0 but greater than or equal to–2.5 at either the lumbar spine or femoral site) and at mild to moderate risk of fracture. Other clinical criteria associated with T-score were considered: advanced age, early menopause (age < 45 years), low body weight, current smoking habit, history of fragility fracture in a first-degree relative. Exclusion criteria included menopause induced by prior chemotherapy or any other drug therapy, metastatic disease, recent hormonal treatment, previous hip (HP) fractures or protheses, known bone metabolism disorder, non-treated hypocalcemia, hypercalcemia, previous treatment with selective estrogen receptor modulators (SERMs), hormone-replacement therapy (HRT) or BPs, liver or renal dysfunction. Height, weight, age and medical history were recorded. The primary end-point was to investigate changes in lumbar spine (LS) and femoral neck (FN) BMD at baseline (T0) and after 24 months (T24). Another secondary end-point was to examine risedronate effect on health-related quality of life (HRQoL) using the short-form healthy survey (SF-36). Women were randomly assigned to receive anastrozole plus oral risedronate or anastrozole alone. All enrolled women received supplements of calcium (1,000 mg/day) and vitamin D (800 IU/day). Anastrozole (Arimidex™; AstraZeneca, London, UK) was given at a dosage of 1 mg/day while oral risedronate (Actonel; Sanofi-Aventis, Paris, France) was given at 35 mg/week. Because oral bisphosphonates are poorly absorbed and for the potential gastrointestinal toxicity, such as esophagitis and esophageal ulcers or erosions, we recommended women to take risedronate early in the morning before taking any food, drink or medicines, remaining upright for at least 30 min (Crandall, 2001; Markopoulos et al., 2010). In our study risedronate therapy was carefully planned and appropriate preventive measures were taken to minimize the risk of developing osteonecrosis of the jaw (Papapetrou, 2009; Silverman and Landesberg, 2009). These include a complete dental examination with preventive dentistry before initiating risedronate, avoiding invasive dental procedures whenever possible, and recommending our women to maintain an excellent oral hygiene (Diel et al., 2007). The BMD was measured at both the LS and FN at baseline and 24 months later (T24) by dual energy x-ray adsorptiometry (DXA) by the same operator and the same densitomer (DXA QDR 1000; Hologic, Waltham, MA, USA). T-scores were determined according to the World Health Organization (WHO) definition as standard deviation (SD) units from the mean BMD of 25-year-old healthy women (Cohen, 2010). Furthermore, women underwent lateral radiographs and digital computerized morphometry (DCM) of thoracic and lumbar spine at T0 and T24. Vertebral fractures were defined as a decrease of more than or equal to 20% in any of vertebral heights (anterior, central, and posterior) (Pedrazzoni et al., 2003). Finally, we administered to the women at each visit the SF-36. This validated questionnaire was used to measure the HRQoL, being the most popular generic health status measure due to its comprehensiveness, shortness and high levels of reliability and validity. The SF-36 contains 36 questions, which take on average 10 min to be answered; it includes eight health concepts: physical functioning (PF), role limitations due to physical problems (RP), bodily pain (BP), general health perceptions (GH), vitality (VT), social functioning (SF), role limitations due to emotional problems (RE) and mental health (MH). Each concept is assessed using a multi-item scale; item scores are summed for each scale and transformed on a scale of 0 to 100, with 0 and 100 corresponding to the poorest and optimal health statuses, respectively. The eight scores design a health profile which is a useful and intuitive tool to describe the HRQoL of a group of individuals or a population. In a second step it is possible to calculate two summary measures which reasume the two major domains of the SF-36: the physical component summary (PCS-36) and the mental component summary (MCS-36) measure. These indexes have the advantage of being easily used, keeping the good properties of the SF-36 and reducing the number of statistical tests necessary for the eight SF-36 scales; they allow a better interpretation of the results (Jenkinson et al., 1993; Liberman et al., 1995; Ware et al., 1995). In this study, SF-36 was applied by one of the investigators as an interview at baseline and at 84 follow-up visits, also offering help by reading and explaining the questions if necessary. The incidence of adverse events and compliance to treatment were recorded at follow-up visits during the study. Data are expressed as mean ± SD or percentage changes from baseline. Changes from baseline in BMD and SF-36 scores were evaluated in both groups of women using a paired *t-*test while unpaired *t*-test was used to examine the change from baseline to 24 months in BMD between the A+R and A groups. A *P*-value of less than 0.05 was considered significant.

## Results

A total of 84 women entered the study; randomly among them 42 women were assigned to receive anastrozole + risedronate (group A+R) and 42 to receive anastrozole alone (group A). A total of 13 women were lost during follow-up, therefore 36 women of group A+R and 35 women of group A completed the study (Figure 1). Baseline characteristics and demographics were similar among the two groups, baseline characteristics are summarized in Table 1.

**Table 1:** Baseline characteristics of women of group A+R (treated with anastrozole plus oral risedronate) and of group A (anastrozole alone).

**Characteristics**	**Group A+R**	**Group A**	*p*-value^*^
N°	42	42	
mean age ± SD	55.7 ± 6.4	56.1 ± 6.3	0.774
height, (cm) mean ± SD	159 ± 8	162 ± 9	0.110
weight, (Kg) mean ± SD	58.0 ± 11.2	60.0 ± 6.3	0.316
BMI, (Kg/m^2^), mean ± SD	23.4 ± 2.6	24.0 ± 2.7	0.303
Mean T-score of lumbar spine	−2.04 ± 0.43	−2.06 ± 0.46	0.838
Mean T-score of femoral neck	−1.80 ± 0.69	−1.98 ± 0.37	0.140

* *p value by sample T-test*.

**Figure 2:**
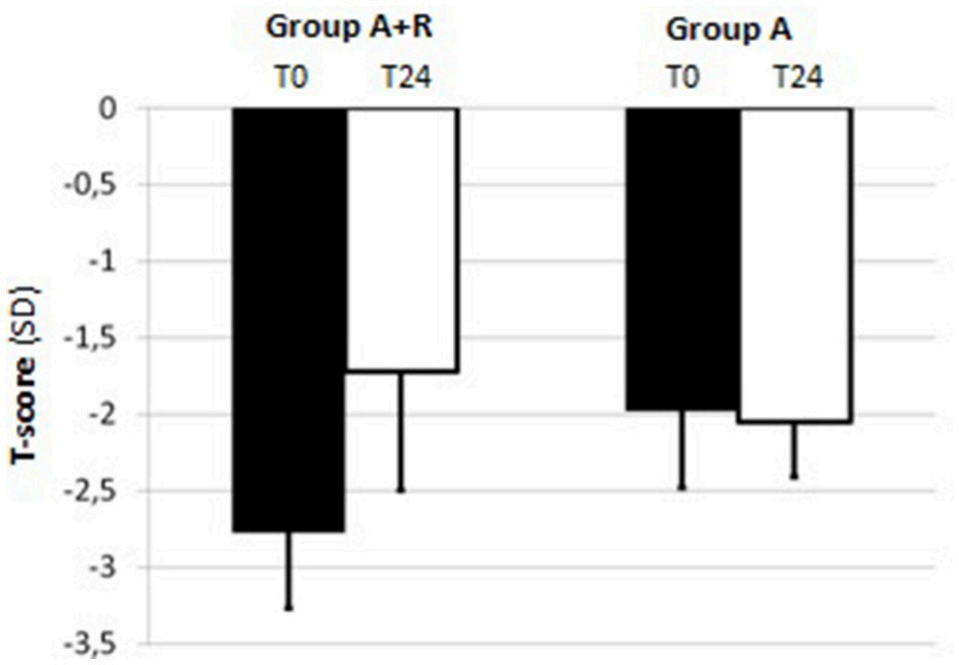
Mean T-score of lumbar spine (LS) at baseline (T0) and at the end of the study (T24) in group A+R (treated with anastrozole plus oral risedronate) and in group A (anastrozole alone).

**Figure 3:**
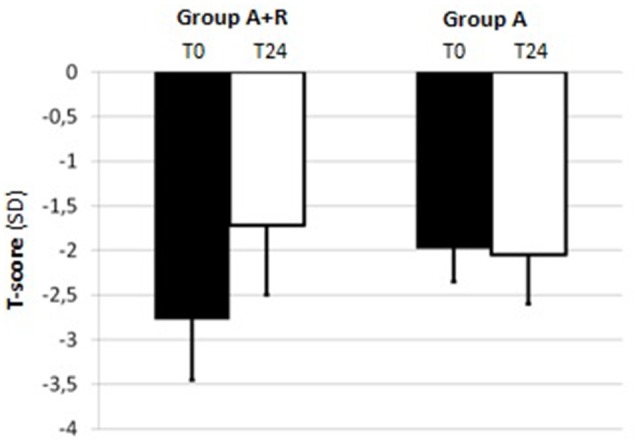
Mean T-score of femoral neck (FN) at baseline (T0) and at the end of the study (T24) in group A+R (treated with anastrozole plus oral risedronate) and in group A (anastrozole alone).

For BMD measurements, mean LS T-score was –2.04 ± 0.43 and mean FN T-score was –1.80 ± 0.69 at baseline in A+R group; in group A mean LS and FN T-score were –2.06 ± 0.46 and –1.98 ± 0.37, respectively (Table 1). The group A+R has showed a significant increase in T-score from baseline to 24 months with an estimated percentage change for LS of +6.86% (*p* < 0.05) and for FN of +2.8% (*p* < 0.05). From T0 to T24 women of group A had a statistically significant rate of bone loss estimated at –4.8% (*p* < 0.05) in LS T-score and a mean significant decrease in FN T-score of –3.5% (*p* < 0.05). Therefore, at 24 month follow-up, T-score BMD was significantly different for group A+R compared with group A both at the lumbar spine –1.9 ± 0.49 vs. –2.16 ± 0.51 (*p* = 0.04) and at femoral neck –1.72 ± 0.78 vs. –2.05 ± 0.36 (*p* = 0.04). Among A+R women, only 2 (5.2%) became osteoporotic, whereas 12 (46%) in group A moved to the osteoporotic BMD region. Mean percentage changes in LS and FN BMD throughout the study period in both groups are reported in Figures 2, 3.

At baseline, 4 of the enrolled women of group A+R and 2 of group A showed vertebral a traumatic fractures; at T24 none of group A+R occurred in new fragility fractures while among women of group A 3 reported a new fragility fracture (only 1 of these had a previous a traumatic fracture).

HRQoL evaluation evidenced that at baseline the SF-36 scores were 39.8 ± 9.7 points in group A and 41.5 ± 12 points in group A+R (*p* > 0.05). At baselinePCS-36 score was 38.2 ± 16 in group A+R and in group A was 36.2 ± 15.3 (*p* > 0.05) whereas MCS-36 score was 37 ± 14 in group A and 35.9 ± 15 in group A+R (*p* > 0.05).

**Figure 4:**
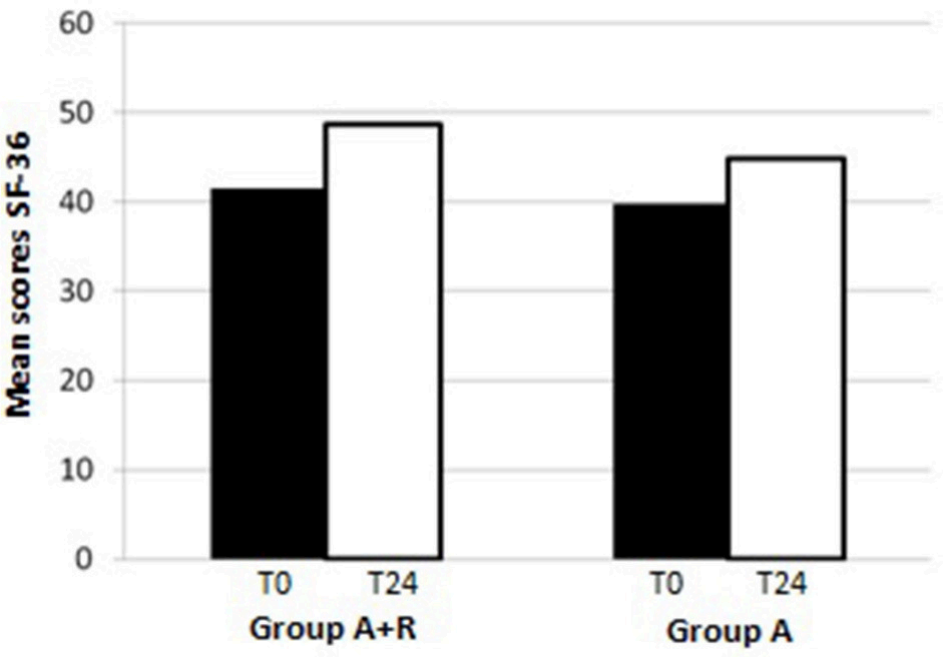
Mean scores of SF-36 at baseline (T0) and after 24 months (T24)in group A+R (treated with anastrozole plus oral risedronate) and in group A (anastrozole alone).

**Figure 5:**
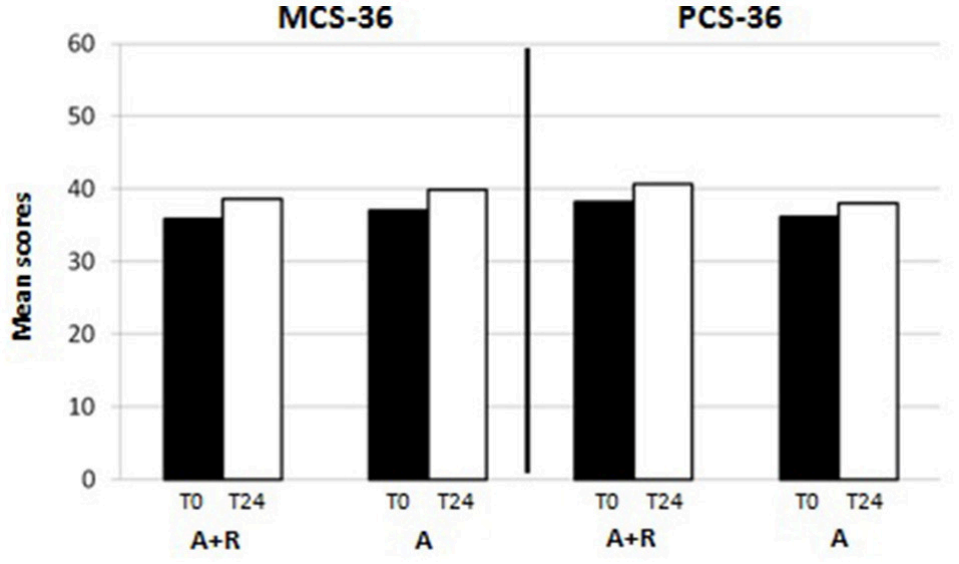
Mean scores of physical component summary (PCS-36) and of mental component summary (MCS-36) at baseline (T0) and after 24 months (T24)in group A+R (treated with anastrozole plus oral risedronate) and in group A (anastrozole alone).

At the end of the study, mean scores in PCS-36 and MCS-36 were, respectively, 40.7 ± 16 and 38.6 ± 16 in group A+R while 38 ± 15 and 39.9 ± 10 in group A. It was evident that after 24 months the group A+R showed an overall significant improvement of health profile: SF-36 score was 48.6 ± 7.3 that is higher than in group A (44.8 ± 6.4; *p* = 0.03) (Figure 4). Specifically, a difference was evident in the physical component (Figure 5): there was a higher positive change in women who were treated with A+R (+6.5%) than in group A in which this percentage change was +5.0%. In MCS-36 percentage changes observed were +7.5% and+7.9% in group A+R and group A, respectively (Figure 5).

Treatment was generally well tolerated and adverse events were comparable among groups, having similar frequency. Specifically, 1 patient of group A+R and 2 of group A stopped the treatment for allergic skin reaction and 2 of the A+R for upper gastrointestinal tract symptoms (Figure 1). Other common transient events were mild gastrointestinal tract symptoms such as abdominal pain, indigestion, nausea, diarrhea, and constipation (8 in group A+R and 2 in group A, respectively). Moreover, 21 women of group A referred musculoskeletal symptoms: 16 women reported arthralgia, 7 myalgia, 4 arthritis, 2 tendinitis and 3 polyalgic syndrome. No case of osteonecrosis of the jaw was observed in any patient on risedronate treatment during the study period. Compliance to treatment was confirmed during the interview in each patient's visit; occasionally, missed tablets of risedronate were reported by less than 5% of the women under study treatment.

## Discussion

Our study, aimed at evaluating the effects of risedronate plus adjuvant AIs therapy on bone health and health status in osteopenic postmenopausal early breast cancer women, demonstrates the usefulness of bisphosphonates in inducing a significant improvement of both lumbar—femoral BMD and HRQoL.

The majority of breast cancers are considered hormone-sensitive due to the presence of estrogen and/or progesterone receptors; for this reason therapeutic strategies interfering with hormone-mediated tumorgenesis have become a cornerstone of breast cancer management paradigm.

The effect of anastrozole and risedronate in menopausal women affected by breast cancer may be also intended as synergistic suggesting to consider it as an interesting clinical management strategy. Moreover, we could speculate that both drugs may act also inhibiting cytokines pathways such as IL6R/IL6-Notch pathways switches and IL2 and/or increasing levels of proinflammatory cytokines such as IFN-gamma, IL-12, and decreasing levels of IL-4, IL-10 secretion. On the other hand, risedronate may be effective for MMP9-expressing AS by targeting immunosuppressive cells such as M2 macrophage and could increase osteoprotegerin and reduce RANKL and IL-1beta levels.

Comparing aromatase inhibitor therapy with tamoxifen in postmenopausal women with estrogen receptor-positive early breast cancer, it was demonstrated a superior efficacy for AIs treatment; this is quickly becoming the gold standard method. However, the residual estrogens are depleted by AIs treatment and this therapy is frequently associated with rapid bone loss and increased fracture risk.

The first study showing a decrement of BMD in anastrozole-treated women was the prospective bone sub-protocol of the ATAC trial (Marra et al., 2013) in which it was demonstrated a bone loss of –3.87% at LS BMD and –3.92% total HP BMD and a significantly higher incidence of fracture using anastrozole (7.1%) than using tamoxifen (4.4%) after 2 years' therapy (Ware et al., 1998; Arimidex, 2006). In recent years some studies have used bisphosphonates in order to avoid aromatase inhibitor-associated bone loss in osteoporotic postmenopausal early breast cancer women. Greenspan et al. (2008) examined positive effect of risedronate in women with or without AIs therapy: after 2 years, in risedronate + AIs group, BMD decreased by –2.4% at spine and was stable at the hip while women on placebo plus AIs had a mean decrease in BMD of –4.8% at the spine and –2.8% at the total hip; in the placebo without AIs group, BMD was stable at the spine, and had a 1.2% loss at the total hip and in risedronate without AIs group showed an improvement in BMD of +2.2% at the total hip (Howell et al., 2005). The SABRE (Study of Anastrozole with the Bisphosphonate Risedronate) demonstrated that in postmenopausal women with breast cancer who are receiving adjuvant anastrozole and having risk of fragility fracture, the use of risedronate led to a 1–3% increase in LS BMD and a 1–2% increase in total HP BMD during a period of 24 months (Van Poznak et al., 2010). Therefore, the beneficial effect of bisphosphonates in reducing bone loss in women affected by osteoporosis has been well described. Instead, the use of bisphosphonates in osteopenic postmenopausal early breast cancer women in AIs therapy is still under debate; in fact, in this case the medical community suggests general preventive measures such as a healthy lifestyle and an adequate intake of calcium and vitamin D. In this context our study could be useful, since it evaluates the use of bisphosphonates as supportive therapy to prevent osteoporosis.

Favorable effects of bisphosphonates on BMD in osteopenic postmenopausal early breast cancer women receiving anastrozole were shown by Markopoulos study (Markopoulos et al., 2010). Women were stratified into those with normal BMD or mild osteopenia (T>–2) receiving anastozole-only (A) and women with mild or severe osteopenia (T≤–2) or osteoporosis (T<–2.5) receiving anastrozole and per os risedronate (A+R). Depending on age of treatment initiation, women were grouped into two age cohorts, above and below 65 years. Among women receiving A-only, women ≤65 years were more likely to have a decrease in LS-BMD than older ones. HP-BMD decrease at 12 months was not related to age. In women with mild severe osteopenia or osteoporosis, treated with A+R, no age effect was observed for LS or HP; among women with normal BMD at baseline, the age effect on LS-BMD change was deeper. This study suggested that younger postmenopausal women with normal BMD or mild osteopenia receiving A-only have an increased risk of bone loss in LS. Among women with mild severe osteopenia or osteoporosis treated with A+R, 12 months LS or HP BMD variations were configured regardless of age group.

Our results demonstrate that the addition of 35 mg oral risedronate weekly to anastrozole improves BMD both at lumbar spine (+6.8%) and at femoral neck (+2.8%) after 24 months in osteopenic postmenopausal early breast cancer women; instead, in osteopenic women who receive anastrozole alone a significant bone loss (–4.5 and –3.5% at spine and femoral neck, respectively) is shown. We observed, in fact, that 46% of osteopenic women developed osteoporosis after 2 years of anastrozole treatment. Our findings suggest, therefore, that risedronate may be used in order to prevent further bone loss in women with mild to moderate osteopenia who have to start adjuvant therapy with AIs in breast cancer.

Major trials have established that AIs improve both disease-free survival and HRQoL and their use as adjuvant endocrine therapy for postmenopausal women with HR+ breast cancer has been steadily increasing. Infact, AIs are also an appealing alternative to tamoxifen because despite their adverse events associated with the menopause-related decline in estrogen level, their use has never been associated with the rare, yet serious, adverse events reported using tamoxifen (e.g., 194 endometrial cancer and venous thromboembolism) (Howell et al., 2005). It should, however, be emphasized that AIs, by reducing plasma estrogens in postmenopausal women, exacerbate bone loss and musculoskeletal symptoms such as arthralgia, myalgia, joint symptoms and polyalgic syndrome. A significant association between BMD values and musculoskeletal symptoms was reported by Muslimani et al. (2009); in this study, it was evidenced that women on AIs developing osteoporosis were at increased risk of musculoskeletal symptoms and bone fracture. However, it was demonstrated that the use of bisphosphonates, in postmenopausal early breast cancer women in therapy with AIs, is associated with adjunctive analgesic benefits, offering these women some relief from arthralgia (Boonen et al., 2004; Pavlakis et al., 2005). In our experience, women treated with anastrazole evidenced severe musculoskeletal symptoms, such as arthralgia, myalgia and arthritis, whereas none of group A+R referred pain of musculoskeletal system. Despite this, little attention has been paid so far to the effect of the use of bisphosphonates on quality of life of these women.

Infact, the novelty of our study is the evaluation of the effect of risedronate addition to adjuvant therapy with AIs on HRQoL. Using SF-36 questionnaire, we observed there was an overall improvement in whole health profile with a slightly higher improvement of physical component in women receiving A+R than in anastrozole group. In fact, the women of this group judged their daily activities (bath, dress, both walk and climb) fairly good; moreover, they reported reduction of pain. These data are quite important since in such women quality of life is an important part of treatment beyond the efficacy indicators.

After all, BPs can play a direct antitumor activity with an added beneficial effect; specifically, it was demonstrated that risedronate and its structural analogs inhibit tumor cell invasion of breast carcinoma cells both *in vitro* and *in vivo*. Therefore, in the cases of women cancers that frequently metastasize to bone, BPs may be used as agents for the prophylactic treatment (Boissier et al., 2000; Fournier et al., 2010).

Moreover, tolerability of administration of risedronate and anastrozole in our study was good with few women showing gastrointestinal symptoms and skin reactions. Overall, the safety profiles of anastrozole and risedronate were consistent with those observed in previous clinical studies (White and Perry, 2003; Yamamoto and Iwase, 2008; Briot et al., 2010). No case of ONJ was observed in any patient on risedronate treatment.

In conclusion, women with osteopenic BMD have considerable risk of developing osteoporosis during the first 2 years of treatment with anastrozole; preventive measures such as healthy lifestyle and daily supplements of calcium and vitamin D alone seem to be insufficient in holding their bones healthy. In our experience, the addition of oral risedronate in postmenopausal breast cancer women in mild to moderate risk region (–1<T-score<–2.5) receiving adjuvant anastrozole gives a significant increase in BMD levels, thus having a very low risk to develop osteoporosis. Moreover, treating women who are on aromatase inhibitors with bisphosphonates also has a significant impact on the patient's quality of life.

Therefore, we suggest the combination of anastrozole plus oral risedronate in order to prevent aromatase inhibitors-related bone loss, not only in postmenopausal breast cancer women at high-risk of fractures, but also for women at mild-moderate risk.

## Author contributions

GAL, VM, RP, AnP, IV, RL, FF, AgP, and MR: conceived the study, participated in its design. MS, FS, FrM, AV, FiM, LM, MG, LC, GCi, and GT: contributed to the conception and design. GAL, VM, GM, and AM: wrote manuscript. AM, MPM, GCa, MM, GL, and GM: drafted the article and revised it critically for important intellectual content; GM and AM: final approval of the version to be published. All authors read and approved the final manuscript.

### Conflict of interest statement

The authors declare that the research was conducted in the absence of any commercial or financial relationships that could be construed as a potential conflict of interest. The reviewer MD declared a shared affiliation, with no collaboration, with several of the authors, VM, GM, IV, MR, MM, AM, to the handling Editor.
